# Innovations in the Treatment of Antibiotic-Resistant Infections in Internal Medicine

**DOI:** 10.7759/cureus.107208

**Published:** 2026-04-17

**Authors:** Sofia Almeida, Pedro M Neves, Vital Da Silva Domingues

**Affiliations:** 1 Internal Medicine, Unidade Local de Saúde de Santo António, Porto, PRT

**Keywords:** antimicrobial resistance, clinical protocols, internal medicine, multidrug-resistant infections, novel antibiotics, stewardship

## Abstract

Antimicrobial resistance (AMR) is one of the major contemporary challenges in internal medicine, impairing the effectiveness of empirical antibiotic regimens and increasing mortality, hospital length of stay, and healthcare costs. The rising prevalence of multidrug-resistant Gram-negative organisms calls for a clinical response grounded in therapeutic innovation, antimicrobial rationalisation, and institutional reorganisation. We conducted a narrative review of the literature from 2020 to 2025, integrating recommendations from international guidelines, including the Infectious Diseases Society of America (IDSA) and the European Society of Clinical Microbiology and Infectious Diseases (ESCMID), epidemiological data from the European Centre for Disease Prevention and Control (ECDC) and the Portuguese PPCIRA (Programa de Prevenção e Controlo de Infeções e de Resistência aos Antimicrobianos) programme, and institutional implementation examples from Portuguese hospitals. Several newly approved antibiotics were identified with documented efficacy against resistant pathogens (e.g., ceftazidime-avibactam, meropenem-vaborbactam, and cefiderocol), along with promising therapeutic approaches such as bacteriophage therapy and immunomodulation. Clinical decision support tools (algorithms, dashboards, checklists) and institutional models demonstrated positive impact on key indicators such as time to active therapy, de-escalation rates, and reduced use of reserve antibiotics. Managing resistant infections in internal medicine requires an integrated, patient-centred approach supported by updated evidence. Internists must take a strategic role in therapeutic decision-making, clinical governance, and the implementation of tailored protocols, actively contributing to AMR containment and improved clinical outcomes.

## Introduction and background

Antimicrobial resistance (AMR) is recognised by the World Health Organization as one of the 10 leading threats to global public health, with widespread impact across both hospital and community care [1,2]. In Europe, it is estimated that more than 35,000 deaths annually are directly associated with infections caused by resistant microorganisms [3].

In Portugal, this phenomenon is particularly concerning, with one of the highest age-adjusted mortality rates from multidrug-resistant infections in Western Europe [2].

Within internal medicine departments, where patients with multiple comorbidities, immunosuppression, invasive devices, and prolonged hospitalisations are concentrated, AMR represents a constant challenge to therapeutic effectiveness [2,4]. The increasing prevalence of carbapenemase-producing *Enterobacteriaceae*, *Pseudomonas aeruginosa* with multiple resistance mechanisms, and resistant *Acinetobacter baumannii* necessitates continuous revision of empirical treatment algorithms [5,6].

In this context, the clinical response cannot be limited to the introduction of new antibiotics.

A multidimensional approach is required, integrating (1) the judicious use of available antimicrobials [7,8], (2) the implementation of antimicrobial stewardship programs [9], (3) the adoption of clinical protocols tailored to local resistance profiles, and (4) the exploration of innovative strategies such as bacteriophage therapy or immunomodulation [10,11].

Against this backdrop, the internist occupies a strategic position. Close involvement with hospitalised patients and a cross-system understanding of pathophysiology confer a central role in the early detection, management, and reassessment of resistant infections [4].

Their work should be coordinated with microbiologists, clinical pharmacists, infectious disease specialists, and therapeutic governance teams, promoting shared and evidence-based decision-making.

This article aims to critically review the main recent therapeutic advances in the treatment of infections caused by multidrug-resistant organisms, focusing on their practical applicability in Portuguese hospital settings [4]. Updated epidemiological data, evidence-based therapeutic options, clinical decision-support tools, and examples of institutional protocols with documented impact will be presented. The goal is to provide a practical guide for internists facing the growing complexity of resistant infections [4].

We conducted a narrative review based on a pragmatic literature search to identify clinically relevant evidence on AMR, including international guidelines, epidemiological reports, and relevant clinical studies, published between January 2009 and December 2025, in the PubMed, Scopus, and Web of Science databases.

## Review

Epidemiological landscape and clinical relevance

The epidemiological situation regarding AMR in Portugal reflects the broader European trend, with persistently high levels of resistance among Gram-negative microorganisms [1,3]. According to the European Centre for Disease Prevention and Control (ECDC) 2023 report, *Klebsiella pneumoniae*, *Escherichia coli*, *Pseudomonas aeruginosa*, and *Acinetobacter baumannii* continue to show concerning rates of resistance to last-line antibiotics such as carbapenems and colistin [3]. The increased risk of resistance is linked to a variety of factors, including the inappropriate use of antibiotics combined with the ability of certain microorganisms to acquire resistance mechanisms [1,12].

The most recent national data, published in the Instituto Nacional de Saúde Doutor Ricardo Jorge (INSA) Epidemiological Bulletin (2025), confirm an increase in carbapenem resistance in *K. pneumoniae* from 4.2% in 2015 to 13.0% in 2022, as well as rising resistance to colistin in *P. aeruginosa* and *Acinetobacter* spp. during the same period [13]. Nevertheless, a significant decline has been observed in the frequency of methicillin-resistant *Staphylococcus aureus* (MRSA) (from 46.9% to 25.0%) and in overall multidrug resistance in *P. aeruginosa* [13]. These data, obtained through the European Antimicrobial Resistance Surveillance Network (EARS-Net), include the active participation of the Unidade Local de Saúde de Santo António (ULSSA), reinforcing the validity and representativeness of national surveillance [13].

The exact mechanisms for the decrease in frequency of MRSA are not yet fully understood, although several studies report this trend. Multiple authors hypothesise that the decline is the result of a combination of enhanced infection control and surveillance, bacterial evolution, and strain replacement [14-16].

This landscape has direct clinical consequences. A European study estimated that approximately 25% of hospital infections caused by multidrug-resistant *Enterobacteriaceae* progress to multiorgan failure or death in the context of sepsis [17].

These findings are particularly relevant in internal medicine departments, which care for patients with multiple risk factors, including repeated hospitalisations, recent antibiotic therapy, chronic diseases, invasive devices, and immunosuppression.

In this scenario, up-to-date knowledge of local resistance profiles becomes essential. The internist must be able to recognise early the risk of infection with multidrug-resistant pathogens and incorporate this assessment into the initial therapeutic decision [5,6]. Major international guidelines, such as those from the Infectious Diseases Society of America (IDSA) and the European Society of Clinical Microbiology and Infectious Diseases (ESCMID), recommend systematic review of empirical antibiotic therapy at 48-72 hours, with de-escalation whenever possible and preference for agents with lower ecological impact [5,6].

In Portugal, these recommendations have been progressively integrated into institutional protocols, with demonstrated positive effects on patient safety and the containment of AMR [4]. Continuous microbiological surveillance, local adaptation of therapeutic algorithms, and targeted clinical training are essential tools for an effective and sustainable response [5,6,9].

Therapeutic innovations and value-based strategies

The treatment of infections caused by multidrug-resistant organisms has evolved significantly over the past decade, with the approval of new antibiotics and the development of promising adjunctive strategies [7,8,18,19]. These advances are particularly relevant in internal medicine, where the management of severe infections is frequent and empirical decisions are often made amid microbiological uncertainty.

Among the most relevant antibiotics are β-lactam/β-lactamase inhibitor combinations. Ceftazidime-avibactam is effective against *Klebsiella pneumoniae* carbapenemase (KPC)-producing *Klebsiella pneumoniae* and some OXA-48 strains and is indicated for complicated urinary tract infections, nosocomial pneumonia, and bacteremia [7]. Meropenem-vaborbactam is effective against KPC but does not cover strains producing metallo-β-lactamases (MBLs) [8]. Ceftolozane-tazobactam is another option with documented efficacy in cases of pan-resistant (PDR) *Pseudomonas aeruginosa* [7].

Cefiderocol, a siderophore cephalosporin, represents an innovation in treating infections caused by Gram-negative bacilli with MBLs, including *Pseudomonas aeruginosa* and *Acinetobacter baumannii* [19]. Its active iron-transport uptake mechanism enhances tissue penetration; however, data from the CREDIBLE-CR study indicate higher mortality in *Acinetobacter* infections, requiring careful consideration of its use [19].

Other recent agents, such as lefamulin, delafloxacin, plazomicin, eravacycline, and omadacycline, expand the therapeutic arsenal, particularly for patients with multiple allergies or resistance to conventional regimens, especially in respiratory and soft tissue infections [18].

Beyond antibiotics, innovative therapeutic approaches are emerging. Bacteriophage therapy has shown efficacy in refractory infections, including osteoarticular and prosthetic infections [10].

Antimicrobial peptides and CRISPR-Cas-based technologies are under investigation as methods for selectively eliminating resistant strains [20]. Synergistic combinations, such as tigecycline with colistin or ampicillin-sulbactam, have been effective against PDR *Acinetobacter baumannii* [7].

From an organisational perspective, the “value-based healthcare” (VBHC) model proposes that therapeutic decisions should be guided by outcomes relevant to the patient while simultaneously considering clinical efficacy, ecological impact, and associated costs [21]. In internal medicine, this translates into rational antibiotic use based on indicators such as time to active therapy, de-escalation rates, and reduced reliance on reserve antibiotics.

The integration of clinical decision-support tools, such as dashboards, adaptive algorithms, and artificial intelligence, operationalises this model [22]. Portuguese researchers have developed HAITooL, a real-time surveillance and clinical decision support system that incorporates computational algorithms to support antimicrobial stewardship, which represents an early form of AI-assisted antimicrobial management [22].

Automated surveillance, enhanced by AI, is emerging as a relevant instrument for anticipating resistance patterns and guiding early interventions [23].

In this context, the internist must master not only the pharmacodynamic and pharmacokinetic principles of new agents but also interpret the clinical and organisational data that underpin rational, safe, and effective prescribing.

Antimicrobial stewardship and organisational literacy

Controlling AMR extends beyond selecting the appropriate antibiotic. It requires a structured organisational approach in which the internist plays a decisive role in therapeutic rationalisation, infection control, and patient safety.

Antimicrobial stewardship (AMS) programs have become pillars of the institutional response to AMR [9,22,23]. The IDSA and Society for Healthcare Epidemiology of America (SHEA) guidelines recommend that every hospital maintain an AMS team, including at least one physician trained in infectious diseases, a clinical pharmacist, and access to microbiological and antibiotic consumption data [5,6].

In Portugal, the PPCIRA (Programa de Prevenção e Controlo de Infeções e de Resistência aos Antimicrobianos) program has encouraged the implementation of such teams since 2017, with documented reductions in broad-spectrum antibiotic use, treatment duration, and the incidence of *Clostridioides difficile* infections [4].

However, the effectiveness of the AMS program does not depend solely on its formal existence. It requires an institutional culture of therapeutic literacy that enables clinicians to interpret local resistance profiles, adhere to protocols, audit prescriptions, and monitor performance indicators [9,22,23].

Given their cross-disciplinary practice and proximity to complex patients, internists should be active agents in this dynamic.

Recent studies highlight the importance of multidisciplinary educational interventions, internal awareness campaigns, and systematic clinical audits as effective measures to improve adherence to rational prescribing [23].

Strengthening continuing education in clinical microbiology, pharmacodynamics, and prudent antibiotic use is considered essential.

These systems should be accompanied by regular interprofessional training and clinical meetings with shared decision-making, fostering an organisational culture of therapeutic safety [6].

Quality management models such as the PDCA (Plan-Do-Check-Act) cycle allow monitoring of key indicators, including time to active antibiotic therapy, de-escalation rate, and proportion of reserve antibiotics, and have documented success [24].

Additionally, the incorporation of tools based on artificial intelligence and machine learning enables the anticipation of resistance patterns while supporting therapeutic decisions based on clinical and microbiological variables [23].

For these tools to be effective, three pillars are required: clinical leadership, cross-disciplinary training, and an organisational environment oriented towards therapeutic governance [23]. Internists must be prepared not only to prescribe but also to actively manage antimicrobial resources, aligning with the institution’s clinical, safety, and sustainability goals [9].

Clinical application and reference protocols

The operationalisation of effective strategies to contain AMR depends on the existence of clear clinical protocols tailored to local epidemiological profiles and integrated into care pathways [9]. Internal medicine, given its cross-cutting role in managing complex patients, should lead or co-lead this implementation.

The Centro Hospitalar Universitário de Santo António (CHUdSA) is likewise one of the participating centres in the national EARS-Net surveillance system, with data integrated into the INSA national report for 2023 [4]. Although formal publications on its internal protocols are not yet available, the institution’s systematic contribution to national resistance data, particularly regarding *Klebsiella pneumoniae*, *Acinetobacter* spp. and *P. aeruginosa*, is a relevant step towards building evidence-based local strategies. In this context, the internist should actively participate in developing, monitoring, and updating such protocols.

**Figure 1:**
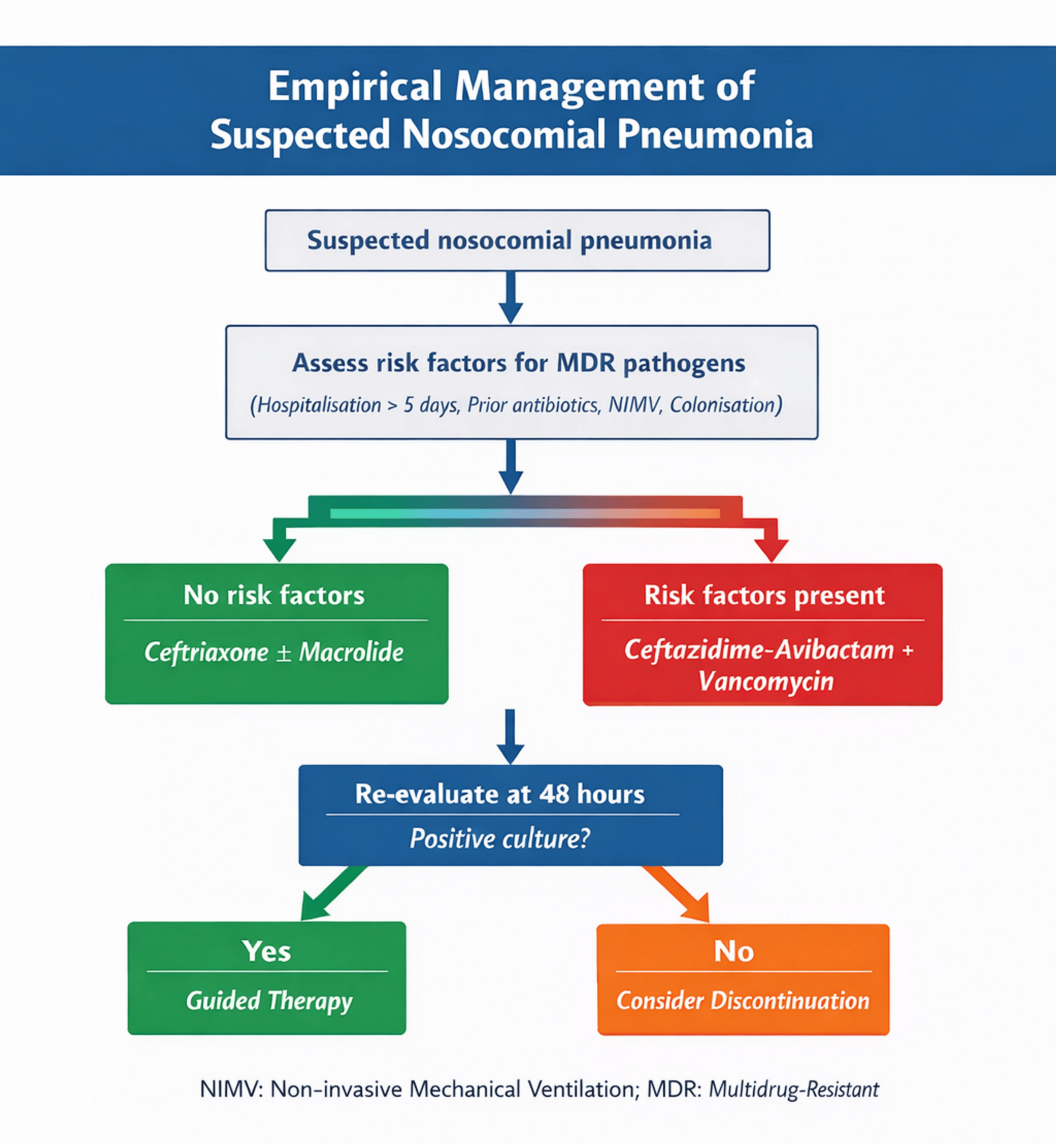
Simplified therapeutic flowchart for empirical decision-making in nosocomial pneumonia based on risk factors for multidrug-resistant pathogens.

These national best practices are complemented by structured tools. Table 1 presents the innovative antibiotics and their respective indications [7,8,18,19], and Table 2 shows the performance indicators in stewardship programs. Figure 1 presents an empirical flowchart adapted to nosocomial pneumonia.

**Table 1:** Newly approved antibiotics (2020-2024) and their respective indications. KPC: Klebsiella pneumoniae carbapenemase; OXA-48: oxacillinase type 48; MBL: metallo-β-lactamase; UTI: urinary tract infection.

Drug	Class	Target	Comments
Ceftazidime–avibactam	Cephalosporin + β-lactamase inhibitor	KPC, OXA-48	Ineffective against MBL
Meropenem–vaborbactam	Carbapenem + inhibitor	KPC	Well tolerated
Cefiderocol	Siderophore cephalosporin	MBL, Acinetobacter spp.	Assess increased mortality risk ↑
Delafloxacin	Fluoroquinolone	Pneumonia, UTI	Option for patients with multiple allergies
Plazomicin	Aminoglycoside	Complicated UTIs	Not recommended as monotherapy for sepsis

**Table 2:** Quality indicators and recommended targets for antimicrobial stewardship programmes in Portuguese hospitals. AMS: antimicrobial stewardship (rational use of antimicrobials).

Indicator	Recommended target	Justification
Time to activate antibiotic therapy	<6 hours	Reduces mortality in sepsis
Reassessment at 48-72 hours	≥90% of cases	Encourages de-escalation
Antibiotic de-escalation rate	≥50%	Reduces ecological impact
Use of reserve antibiotics	<10% of total	Containment of resistance
Participation in AMS audits	≥2/year/unit	Continuous improvement

The internist must ensure that empirical antibiotic therapy is initiated effectively, reviewed promptly, and adjusted according to clinical and microbiological criteria, ensuring a rational, safe, and patient-centred approach.

## Conclusions

AMR represents one of the greatest current challenges in internal medicine, combining clinical complexity, organisational pressure, and direct epidemiological implications. Therapeutic innovations, such as new β-lactam/β-lactamase inhibitor combinations, agents like cefiderocol, bacteriophage therapies, and CRISPR-based approaches, represent promising advances, but only if integrated into rational prescribing strategies. National experience shows that the implementation of institutional protocols, clinical dashboards, and stewardship teams has a measurable impact on indicators such as severe sepsis mortality, time to active antibiotic therapy, and de-escalation rates.

Containing AMR does not depend solely on the discovery of new molecules. Above all, it depends on how we use the therapeutic resources available with rationality, rigour, and clinical leadership. Internal medicine possesses the knowledge, scope, and responsibility to lead this response. The internist must assume this role with determination and commitment.

## Appendices

Practical recommendations for the internist

· Initiate empirical antibiotic therapy within the first 60-90 minutes in cases of sepsis or severe infection, based on clinical risk factors and local resistance profiles.

· Systematically reassess therapy at 48-72 hours, integrating microbiological data and clinical evolution, and promote de-escalation whenever possible.

· Use updated institutional protocols, ensuring clear documentation of therapeutic decisions and alignment with antimicrobial stewardship team recommendations.

· Avoid reserve antibiotics outside defined criteria, limiting their use to microbiologically validated indications.

· Monitor key indicators such as time to active therapy, de-escalation rates, treatment duration, and broad-spectrum antibiotic consumption.

· Actively participate in stewardship teams and clinical audits, contributing to institutional therapeutic governance and multidisciplinary team training.

· Foster interprofessional communication, ensuring shared decisions are aligned with the institution’s clinical and strategic objectives.
